# Retinoblastoma-E2F Transcription Factor Interplay Is Essential for Testicular Development and Male Fertility

**DOI:** 10.3389/fendo.2022.903684

**Published:** 2022-05-19

**Authors:** Juho-Antti Mäkelä, Jorma Toppari

**Affiliations:** ^1^Institute of Biomedicine, Research Centre for Integrative Physiology and Pharmacology, University of Turku, Turku, Finland; ^2^Department of Pediatrics, Turku University Hospital, Turku, Finland; ^3^Centre for Population Health Research, University of Turku and Turku University Hospital, Turku, Finland

**Keywords:** retinoblastoma protein, spermatogenesis, testis, E2F transcription factor, germ cell, Sertoli cell

## Abstract

The retinoblastoma (RB) protein family members (pRB, p107 and p130) are key regulators of cell cycle progression, but also play crucial roles in apoptosis, and stem cell self-renewal and differentiation. RB proteins exert their effects through binding to E2F transcription factors, which are essential developmental and physiological regulators of tissue and organ homeostasis. According to the canonical view, phosphorylation of RB results in release of E2Fs and induction of genes needed for progress of the cell cycle. However, there are eight members in the E2F transcription factor family with both activator (E2F1-3a) and repressor (E2F3b–E2F8) roles, highlighting the functional diversity of RB-E2F pathway. In this review article we summarize the data showing that RB-E2F interaction is a key cell-autonomous mechanism responsible for establishment and maintenance of lifelong male fertility. We also review the expression pattern of RB proteins and E2F transcription factors in the testis and male germ cells. The available evidence supports that RB and E2F family members are widely and dynamically expressed in the testis, and they are known to have versatile roles during spermatogenesis. Knowledge of the function and significance of RB-E2F interplay for testicular development and spermatogenesis comes primarily from gene knock-out (KO) studies. Several studies conducted in Sertoli cell-specific *pRB*-KO mice have demonstrated that pRB-mediated inhibition of E2F3 is essential for Sertoli cell functional maturation and cell cycle exit, highlighting that RB-E2F interaction in Sertoli cells is paramount to male fertility. Similarly, ablation of either pRB or E2F1 in the germline results in progressive testicular atrophy due to germline stem cell (GSC) depletion, emphasizing the importance of proper RB-E2F interplay for germline maintenance and lifelong sperm production. In summary, while balanced RB-E2F interplay is essential for cell-autonomous maintenance of GSCs and, the pRB-E2F3 system in Sertoli cells is critical for providing GSC niche thus laying the basis for spermatogenesis.

## RB-E2F Pathway

**Figure 1:**
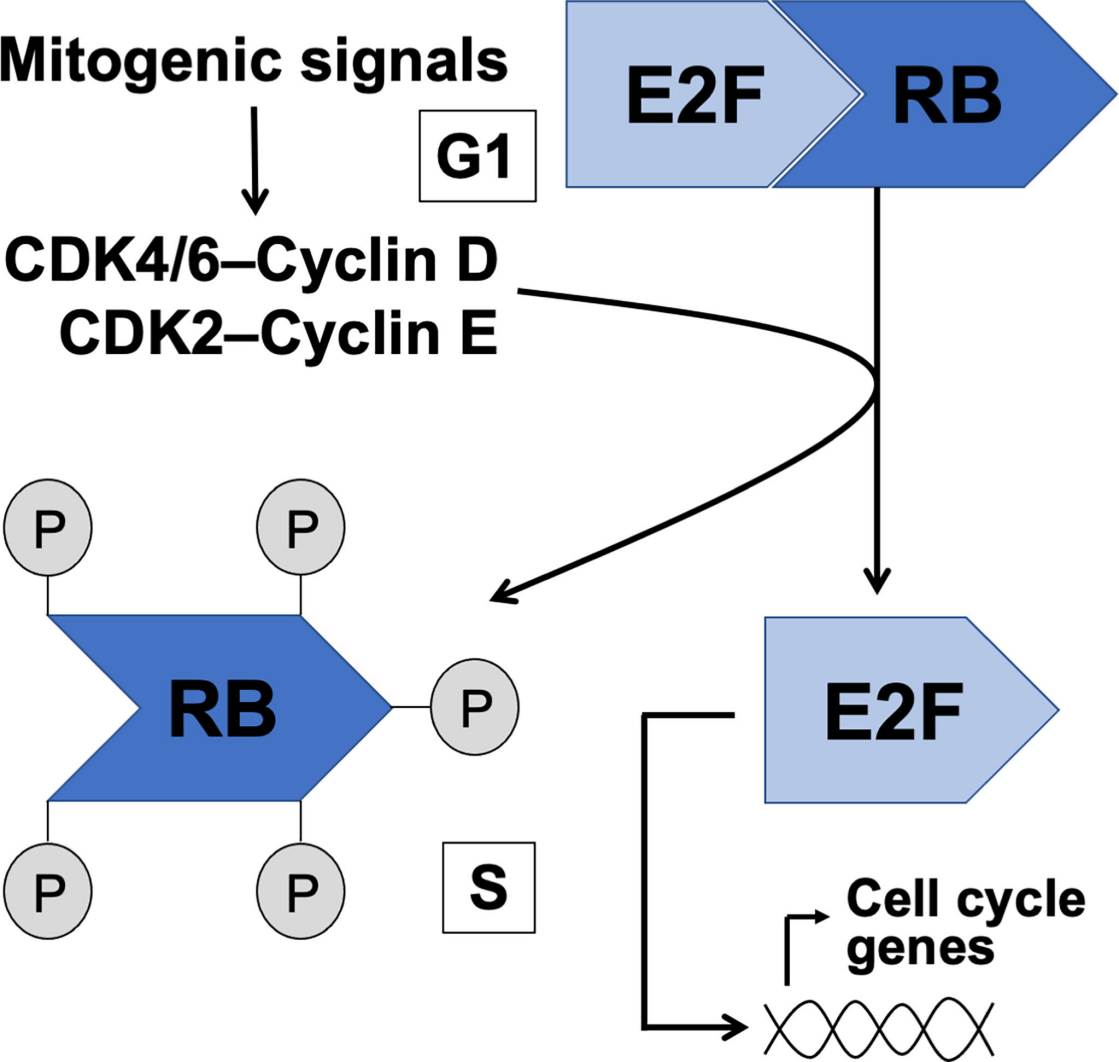
The canonical RB-E2F pathway in control of G1 to S phase transition. Intra- and extracellular mitogenic signals are considered to converge on CDK/cyclin activation resulting in phosphorylation of RB, release of E2F and transcription of S phase genes.

Strict regulation of the cell cycle is critical during testicular development and steady-state spermatogenesis. The mechanisms that define whether a cell stays in the G_1_ state or transits to S phase or G_0_ are ultimately responsible for normal development of any tissue and its function under homeostasis. The G_1_/S transition is controlled by the interaction between retinoblastoma (RB) tumor suppressor proteins and E2F transcription factors. In G_1_ RB family proteins form complexes with E2Fs and various chromatin modifiers to repress E2F activity on the promoters of the genes that are needed to enter the S phase. The function of RB is thus growth-inhibitory and misregulation of RB-E2F interplay is one of the hallmarks of cancer (1). The RB-dependent repression on E2F-driven transcription is relieved upon phosphorylation of RB by cyclin-dependent kinases (CDKs) resulting in cellular growth, DNA synthesis and advancement of the cell cycle (**Figure 1**). It is considered that many different stimuli that affect cell fate decisions are channeled through CDKs to control the phosphorylation status of RB to ultimately control the progress or arrest of the cell cycle at G_1_ (2).

Efficient entry in the S phase depends on numerous phosphorylation events on RB resulting in the dissociation of the RB-E2F complex and activation of E2F transcription factors. While numerous different kinases have a capacity to phosphorylate RB (3), the role of CDK4/6 and CDK2 in G1/S transition is probably the most critical (4). The transcriptional targets of E2F include *cyclin E*, *Cdk1, DNA polymerase-alpha* and *E2f*s themselves (5). While the molecular control of CDK-RB-E2F pathway has been much studied and relatively well understood, the versatility in RB and E2F protein families brings a layer of complexity to the big picture.

The retinoblastoma protein family consists of three proteins (pRB, p107 and p130; also known as RB1, Retinoblastoma-like 1 and Retinoblastoma-like 2 (RBL1-2), respectively) that share significant homology, yet only *pRb* is found frequently mutated in cancer (6). While some degree of functional redundancy in the RB protein family is apparent - especially between p107 and p130, as observed in mouse knock-out studies - the data support an indispensable role for pRB in development and oncogenesis [reviewed in (7)]. Yet, in a physiological context evaluation of such aspects is often irrelevant due to cell-type dependent expression patterns, as we will later see also in the testis. Thus far eight different *E2f* genes (*E2f1-8*) are known, giving rise to nine distinct E2F proteins. E2F1, E2F2 and E2F3A are generally considered as transcriptional activators, whereas E2F3B and E2F4-8 have been assigned transcriptional repressor functions. However, at least in some developmental contexts E2Fs display functional plasticity and may interconvert between repressor and activator functions (8–12). While activator E2Fs are generally considered to function in cell proliferation, repressor E2Fs are involved in cell cycle exit and differentiation (13). However, such clear-cut functional dichotomy in a physiological context barely exists, and the downstream effects of E2F activation are context-dependent. A notable feature in the E2F protein family is functional redundancy, and in many cases two or more *E2f* genes need to be ablated in order to achieve a phenotypic change (14).

RB family proteins are so called pocket proteins that interact widely with different proteins (>300 proteins have been identified as possible binding partners), not just with E2Fs (15, 16). Within the E2F family they show preferences in terms of interaction partners, and while E2F1-3 predominantly associate with pRB, E2F4-5 preferentially interact with p107 or p130 (7). Conversely, E2F6 is an RB-independent transcriptional repressor and instead forms a complex with Polycomb group proteins (17), and E2F7/8-driven transcriptional repression is also independent from CDK-mediated phosphorylation of RBs (18). Notably, the function of RBs goes beyond gatekeeping G_1_/S transition and they play critical roles in quiescent, senescent and differentiating cells by maintaining G_0_/G_1_. Among E2Fs the interaction of E2F1 with pRB is considered unique and even hyperphosphorylation of pRB does not fully preclude this interaction (19, 20). Conspicuously, E2F1 is also the most extensively studied of the E2F family members and future investigations will be required to elucidate the nuances in tissue-, cell- and context-dependent action of different E2Fs.

In addition to its canonical role in control of the cell cycle and proliferation, the RB-E2F pathway has also been implicated in regulation of heterochromatin and chromosome stability, and apoptosis (2), all of which are very relevant to spermatogenesis. A number of studies have shown that increased E2F activity due to RB loss-of-function has an adverse effect on chromosome stability and causes aneuploidy (2, 21). One of the underlying mechanism may be deregulation of the pericentric heterochromatin, an essential chromosomal region for proper segregation during mitosis and meiosis (22, 23). Considering the role of activator E2Fs in cell cycle progression, it is somewhat counterintuitive that increased E2F activity is also able to induce apoptosis *via* DNA damage signaling pathways. According to a model proposed by Dick & Rubin (2013) this involves extensive post-transcriptional modifications in both RB and E2F1 allowing expression of pro-apoptotic genes, but repression of E2F-dependent cell cycle genes (2). All these different functional aspects of RB-E2F pathway make it a key regulator of testicular development and physiology, as will be highlighted in this article.

## Dynamic Expression of RBs and E2Fs in Rodent Testis

### mRNA Expression in Mouse Spermatogenic Cells

**Figure 2:**
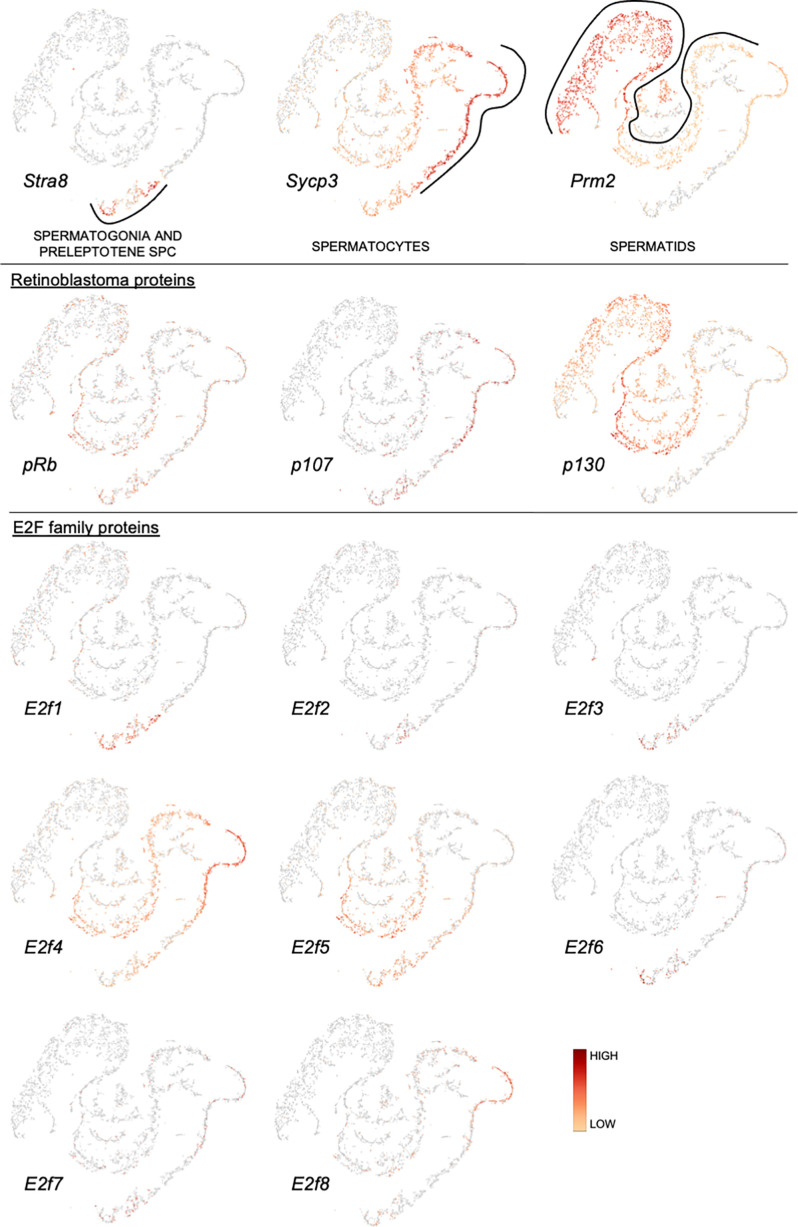
Expression of retinoblastoma protein family and E2F transcription factor family mRNAs along a pseudotime trajectory of adult mouse spermatogenic cells. The data is extracted from Hermann et al. 2018 (26) using Loupe Cell Browser v6.0.0 from 10x Genomics. *Stra8*, *Sycp3* and *Prm2* are included to denote spermatogonia/preleptotene spermatocytes, spermatocytes and spermatids, respectively.

**Figure 3:**
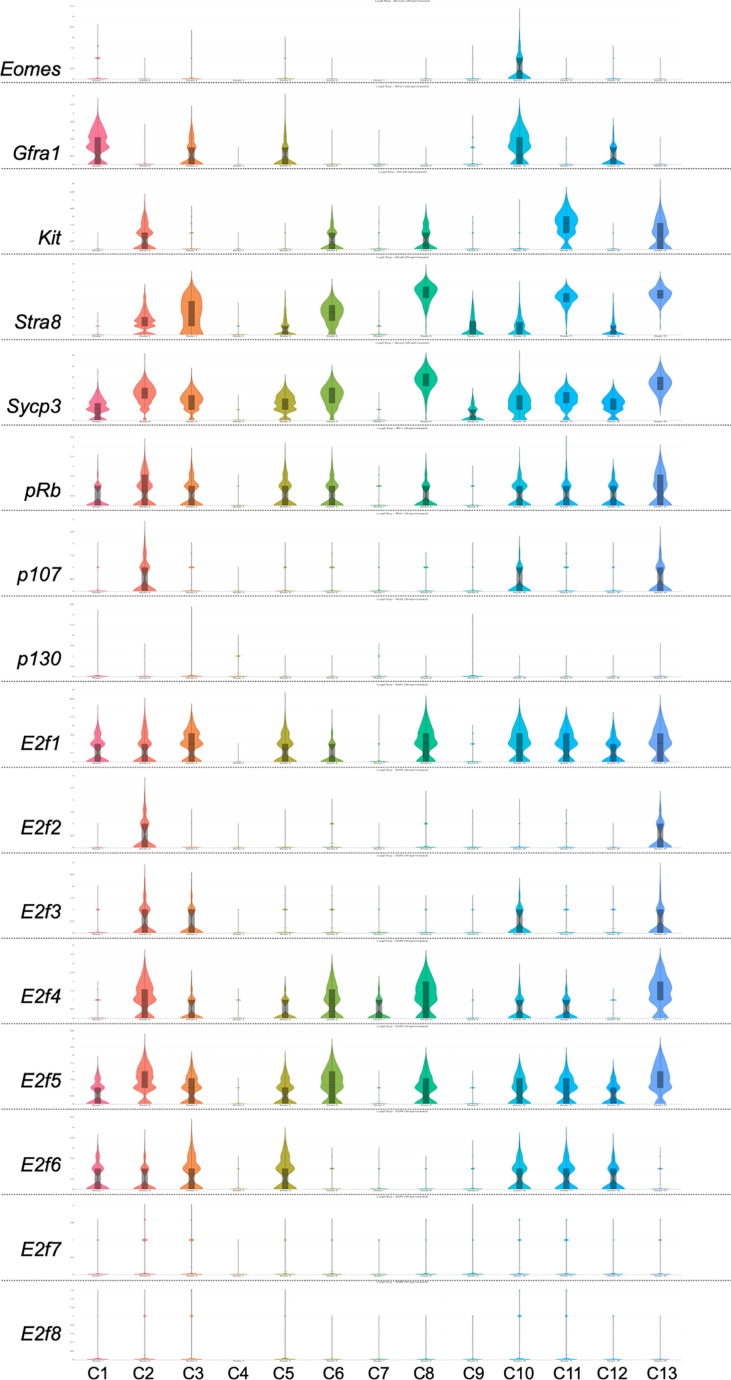
Violin plots displaying the expression of *Eomes, Gfra1, Kit, Stra8, Sycp3, pRb, p107, p130* and *E2f1-E2f8* in spermatogonial cells of an adult mouse. The data is extracted from Hermann et al. 2018 (26) using Loupe Cell Browser v6.0.0 from 10x Genomics. Spermatogonia are divided into clusters 1-13 (C1-13).

**Figure 4:**
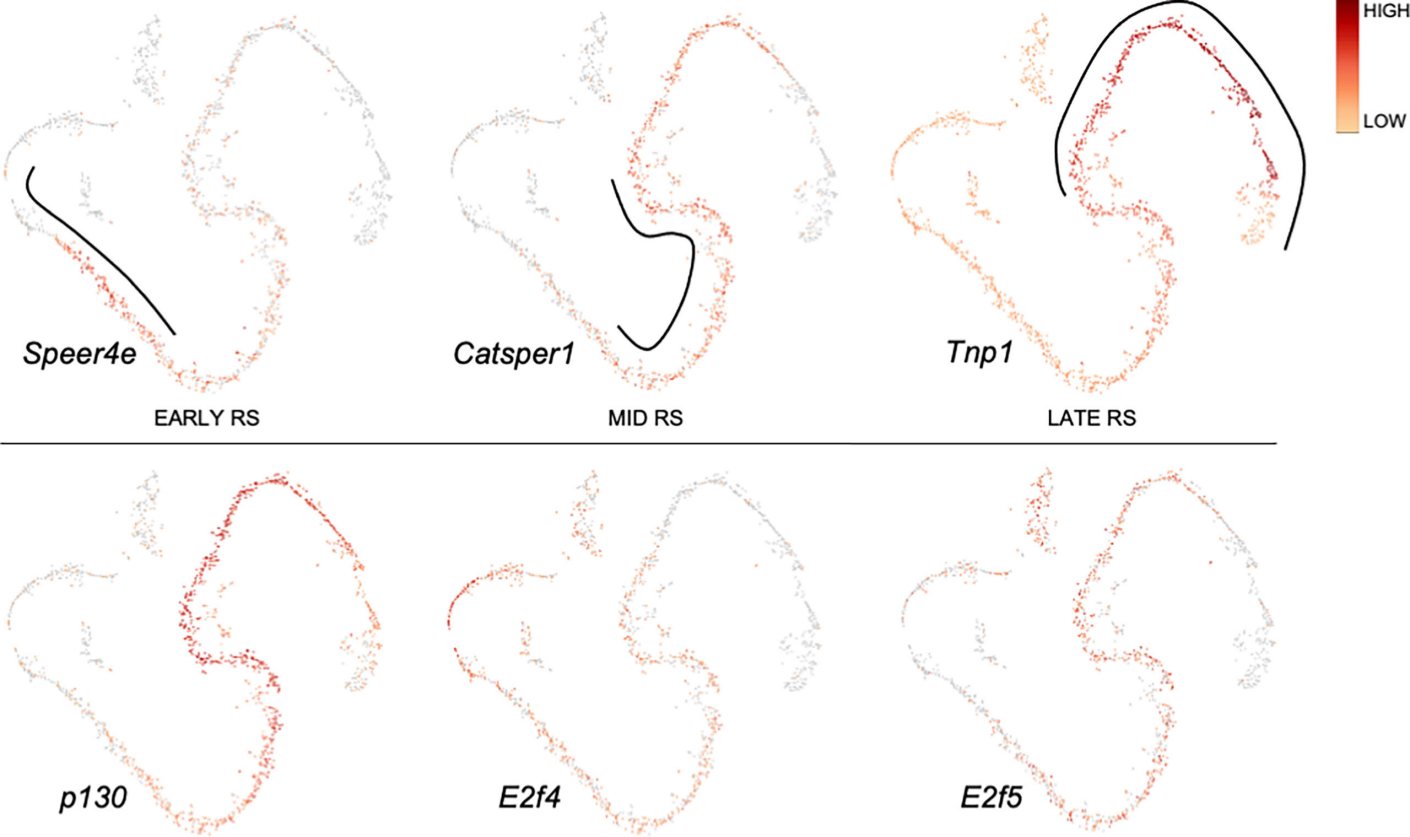
Expression of *p130, E2f4* and *E2f5* along a pseudotime trajectory of sorted mouse round spermatids. The data is extracted from Hermann et al. 2018 (26) using Loupe Cell Browser v6.0.0 from 10x Genomics. Expression for *Speer4e, Catsper1* and *Tnp1* are included to demarcate early, mid and late round spermatids, respectively.

Application of single-cell RNA-sequencing technology (scRNA-seq) for the analysis of gene expression during spermatogenesis has provided a powerful tool to better understand its molecular regulation and identify the genes/pathways involved in it in various organisms (24–32). We took advantage of a previously published adult mouse testis scRNA-seq dataset (26) and Loupe Cell Browser v6.0.0 from 10x Genomics to visualize the distribution of RB and E2F family mRNAs along the entire spermatogenic trajectory (**Figure 2**), or specifically in spermatogonia (**Figure 3**) and round spermatids (**Figure 4**). We chose the dataset of Hermann et al. (2018) because it is the first comprehensive scRNA-seq analysis of adult mouse spermatogenic cells distinguishing 11 cell types (26) and enabling feasible analysis of their gene expression signature. For this analysis spermatogonia were further divided into clusters (1-13; **Figure 3**), with clusters 1, 3, 5 and 10 representing undifferentiated spermatogonia as defined by expression of *Gfra1* (33, 34) and *Eomes* (35, 36), and clusters 2, 3, 6, 8, 11 and 13 representing differentiating spermatogonia with characteristic expression for *Kit* (37, 38)*, Stra8* (39, 40) and *Sycp3* (41, 42). Round spermatids were divided into early, mid and late steps (**Figure 4**) based on expression of *Speer4e*, *Catsper1* and *Tnp1* (26). Besides the entire spermatogenic trajectory (**Figure 2**), we decided to focus on GSCs/spermatogonia (**Figure 3**) because they are the foundation of spermatogenesis, and round spermatids (**Figure 4**) because *p130*, *E2f4* and *E2f5* display intriguing expression patterns in these cells. It should be noted that the data presented in **Figures 2**–**4** are from a previously published dataset (26) and their reliability have not been validated with independent approaches.

#### pRB (Rb1)

*pRB* is expressed rather uniformly throughout spermatogenic differentiation (**Figure 2**), and also in most cell clusters within the spermatogonial compartment (**Figure 3**).

#### p107 (Rbl1)

*p107* is expressed in spermatocytes, and to a lesser extent in early spermatids (**Figure 2**) and spermatogonia (**Figure 3**).

#### p130 (Rbl2)

*p130* is expressed in spermatids (**Figure 2**) with the highest levels in mid-to-late round spermatids (**Figure 4**).

#### E2f1

*E2f1* is expressed in spermatogonia and early spermatocytes (**Figure 2**), which is also supported by RNA *in situ* results (43). *E2f1* expression is rather uniform in all spermatogonial clusters (**Figure 3**) from GSCs (*Eomes/Gfra1*+; clusters 1 and 10) to differentiating spermatogonia (*Kit/Sycp3/Stra8*+; clusters 2, 3, 6, 8, 11 and 13).

#### E2f2

There are very few cells expressing *E2f2* along the spermatogenic trajectory (**Figure 2**).

#### E2f3

*E2f3* is expressed in a limited fashion in spermatogonia and preleptotene spermatocytes (**Figure 2**).

#### E2f4

*E2f4* is expressed in spermatogonia, spermatocytes and round spermatids (**Figure 2**). In spermatogonia (**Figure 3**) the highest *E2f4* levels are seen in clusters 2, 6, 8 and 13 (*Kit/Sycp3/Stra8*+) corresponding to differentiating spermatogonia. In round spermatids *E2f4* levels are highest in early and mid steps (**Figure 4**).

#### E2f5

*E2f5* closely follows the expression pattern of *E2f4* but is clearly less expressed in spermatocytes and early round spermatids (**Figure 2**). Within the round spermatid population the highest levels are seen in mid to late steps (**Figure 4**). Expression in spermatogonial clusters is rather uniform but with highest levels in differentiating spermatogonia (**Figure 3**).

#### E2f6

*E2f6* expression is restricted to spermatogonia and only sporadically detected elsewhere (**Figure 2**). Within the spermatogonial compartment *E2f6* is enriched in clusters that are also positive for *Gfra1* (1, 3, 5 and 10) and thus potential GSCs (**Figure 3**).

#### E2f7-8

Spermatogenic expression for *E2f7* and *E2f8* is low. Late spermatocytes and/or early round spermatids show limited *E2f8* expression (**Figure 2**).

### Protein Expression in Rodent Testis

**Figure 5:**
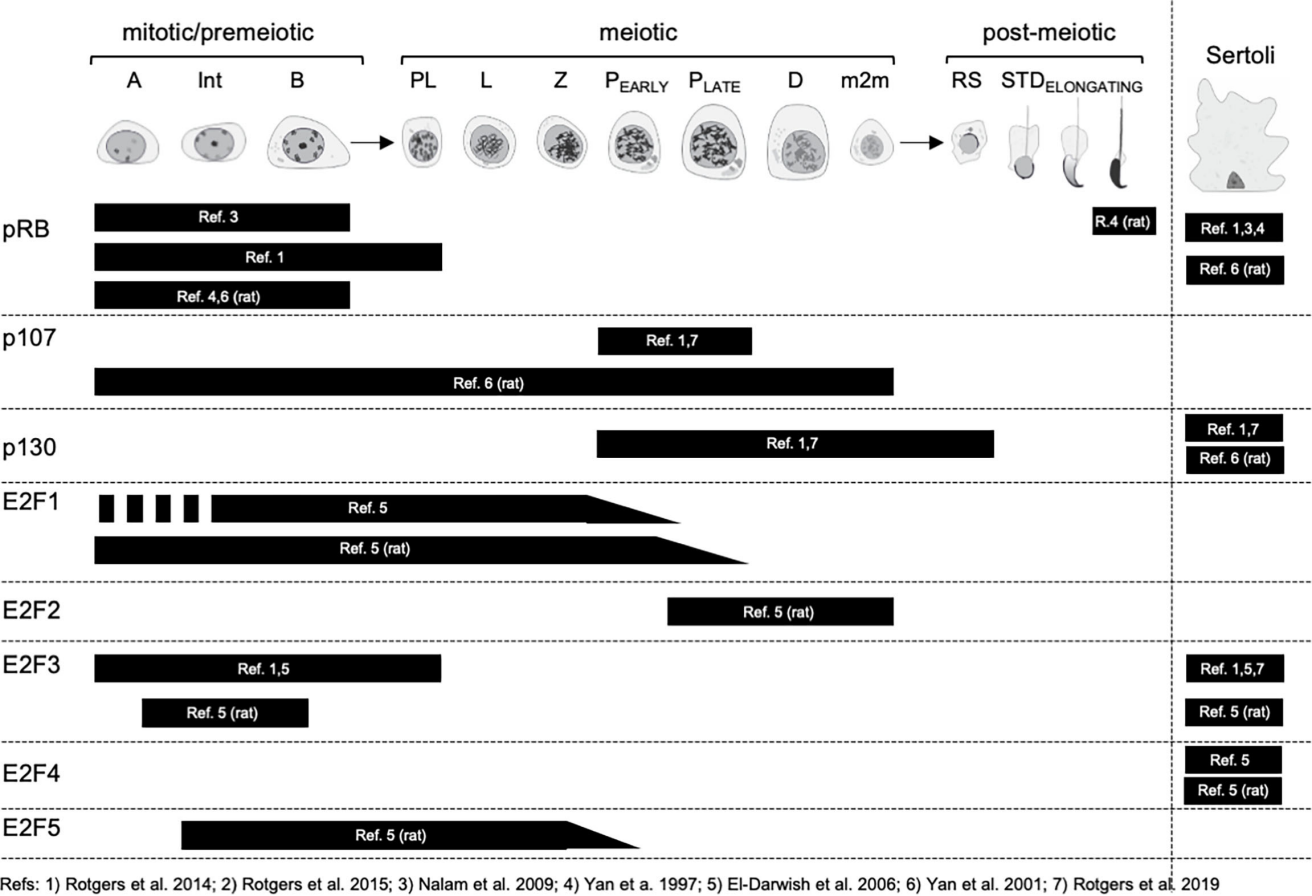
Expression of RB and E2F family proteins in spermatogenic cells and Sertoli cells. Mitotic/premeiotic germ cells are divided into type A (A), type Intermediate (Int) and type B (B) spermatogonia. Meiotic germ cells are presented as preleptotene (Pl), leptotene (L), zygotene (Z), early and late pachytene (P) and diplotene (D) spermatocytes, and secondary spermatocytes, m2m. Post-meiotic germ cells are round spermatids (RS) and elongating spermatids (STD).

The protein expression of all RB and most E2F family members have been studied in a handful of articles. The expression pattern for each protein is summarized in **Figure 5**.

#### pRB (RB1)

In the mouse testis Nalam et al. (2009) reported the staining of pRB in Sertoli cells and spermatogonia (44), while Rotgers et al. (2014) observed the same but extended the staining to preleptotene spermatocytes (45). In the rat testis Yan et al. (2001) confirmed pRB staining in Sertoli cells and spermatogonia but also showed that the highest levels of Sertoli cell pRB in seminiferous epithelial stages VII-VIII (46). The protein expression of pRB in spermatogenic cells is to some extent in conflict with mRNA level findings (**Figure 2**), and suggests that *pRB* mRNA is not translated to protein in post-mitotic male germ cells.

#### p107 (RBL1)

Rotgers et al. (2014 & 2019) reported p107 staining solely in pachytene spermatocytes in adult WT mouse (45), which is in agreement with single-cell RNA-sequencing data (**Figure 2**). In the rat testis p107 was localized to spermatogonia and spermatocytes (46).

#### p130 (RBL2)

In the mouse testis Sertoli cells were found positive for p130 (45, 47). Interestingly, the authors did not mention a weak staining for p130 in spermatocytes and round spermatids that would also be supported by enrichment of *p130* mRNA in these germ cells (**Figure 2**). In the rat testis p130 protein expression is seen in Sertoli cells, Leydig cells and peritubular myoid cells, and excluded from differentiating germ cells (46).

#### E2F1

The expression of E2F1 in the mouse testis has been reported only in one study. El-Darwish et al. (2006) showed the expression in Intermediate to type B spermatogonia and in leptotene to early pachytene spermatocytes (48). The highest levels of E2F1 were observed in stages IX-XI, *i.e* leptotene and zygotene spermatocytes. These findings align with mRNA expression analysis, although leave the protein expression in type A spermatogonia elusive. Based on functional analyses (discussed later in the text) it is probable that E2F1 is expressed at the protein level already in type A spermatogonia. In the rat testis the E2F1 staining differs slightly with type A spermatogonia already expressing it, and the expression extending to mid-to-late pachytene spermatocytes (48).

#### E2F2

El-Darwish et al. (2006) also studied the localization of E2F2 in rodent testis. In the rat testis E2F2 is most highly expressed in pachytene and diplotene spermatocytes of stages VII-XIII, but also in secondary spermatocytes (48). In the mouse the results show abundant E2F2 staining in nearly all mouse testicular cell types, excluding condensing spermatids. Compared to the very restricted pattern of expression in the rat and the scanty mRNA expression in mouse (**Figure 2**) the results concerning the staining in the mouse would require further investigation and validation.

#### E2F3

The data concerning E2F3 expression in the mouse testis is contradictory. While the expression in Sertoli cells is supported by all available data (45, 47, 48), germ cell expression remains somewhat elusive. While El-Darwish et al. (2006) and Rotgers et al. (2014) reported E2F3 staining in spermatogonia and preleptotene spermatocytes (45, 48), which is compatible with mRNA expression (**Figure 2**), Rotgers et al. (2019) did not confirm spermatogonial staining with E2F3a-specific or pan-E2F3 antibodies in the adult mouse testis (47). Isoform unspecific E2F3 antibody, however, did stain spermatogonia in the juvenile testis. In the adult rat testis E2F3 expression was seen in Sertoli cells and spermatogonia (48).

#### E2F4

Strong E2F4 staining is seen in the nuclei of testicular somatic cells: Sertoli cells, Leydig cells and peritubular myoid cells both in mouse and rat (48). The authors speculate though, that only the Sertoli cell staining is specific because parallel staining of *E2f4* KO mouse testis sections show that only Sertoli staining was absent. Interestingly, there is a granular staining for E2F4 from spermatocytes to elongated spermatids both in mouse and rat.

#### E2F5

E2F5 is expressed from type Intermediate spermatogonia to mid-Pachytene spermatocytes in the rat testis (48). To our knowledge E2F5 localization in the mouse testis has not been studied.

As far as we know there are no studies where the localization of E2F6, E2F7 or E2F8 in rodent testis would have been studied.

## Functional Consequences of RB-E2F Pathway Loss-of-Function in the Testis

### pRB Is Essential for Establishment and Maintenance of GSCs

The fact that *pRB*-null mice are embryonically lethal (49–51) has complicated the study of pRB's tissue-specific functions. In the testicular context, however, this limitation has been circumvented by creation of cell type-specific conditional KO mice (44, 45, 52–54) and *ex vivo* culture (55). Of note, *p107*- and *p130*-deficient mice are viable, healthy and fertile (56, 57). We will first focus on the effects of germ cell-specific ablation of pRB and then summarize the role of pRB in Sertoli cells. The available literature indicates that pRB is indispensable for formation and maintenance of germline stem cells (GSCs). Lifelong ability of sperm production depends on GSCs, whose cell fate decisions need to be tightly balanced in order to maintain high and continuous production of sperm throughout the reproductive life span. In theory, GSCs must undergo both self-renewal divisions to sustain the GSC pool and differentiation divisions to give rise to transit-amplifying progenitor spermatogonia that are destined to complete spermatogenesis. Mechanistically it is not entirely clear to what extent these cell fate decisions are cell-autonomous and how/when the molecular cues in the GSC niche microenvironment are incorporated into the regulation of these events. The stem cell capacity in the mouse testis is thought to reside in a subset of undifferentiated spermatogonia (A_undiff_), although in regenerative conditions A_undiff_ display functional plasticity suggesting that the number of potential stem cells markedly exceeds that of steady-state stem cells (58, 59).

Given its role as the gatekeeper of G1/S transition, or quiescence vs. proliferation, pRB is understandably a critical regulator of GSCs. The evidence for this comes from various mouse models where *pRB* has been conditionally deleted from germ cells at different ages: prenatally from primordial germ cells (*pRB*-KO*^Blimp1^*) (53) or ED15.5-17 gonocytes (*pRB*-KO*^Ddx4^*) (52, 54), and postnatally from progenitor-A_undiff_ (*pRB*-KO*^Ngn3^*) (54) and progenitor/differentiating spermatogonia (*pRB*-KO*^Stra8^*) (54). Contrary to its role in somatic stem cells where inactivation of RB family proteins often results in stem cell expansion, increased apoptosis, altered cell fate/differentiation defects, and initiation of cancer (60), pRB-deficient male GSCs have been reported to lose their capacity to self-renew, possibly explaining why no testicular tumors were observed (52, 54). Notably, the differentiation capacity of *pRB*-null germ cells was not affected but young adult males (until 2-3 months of age) were able to sire offspring. However, due to gradual depletion of GSCs they became infertile with age (52, 54).

Despite the fact that pRB is expressed very early in the germ lineage, its absence does not seem to have any effects on fetal germ cells, termed gonocytes (or prospermatogonia), before ED14.5 (53, 55) which is the time when WT gonocytes start to enter mitotic quiescence (61). Recently, Du et al. (2021) discovered that at ED16.5, when practically all control gonocytes had stopped proliferating, the majority of pRB-KO*^Blimp1^* were still engaged in the cell cycle resulting in two-fold higher number of germ cells at the same time point. Subsequently, there is a massive wave of apoptosis in pRB-KO*^Blimp1^* testes that ablates the germline by the time of onset of the first round of spermatogenesis (PND3.5-6.5) (53). Interestingly, in this time window the classical effects of pRB-deficiency are also recapitulated in the germline: mitotic overexpansion followed by increased apoptosis. It is not exactly clear what induces programmed cell death in practically all pRB-KO*^Blimp1^* germ cells perinatally, but there are a number of factors that might contribute to it, including a failure of pRB-KO*^Blimp1^* gonocytes to inhibit the onset of meiosis, a demarcating feature of all fetal male germ cells (53). It has been well documented that the gonocyte population experiences a wave of apoptosis between ED13.5 and ED17.5 (62, 63), which is considered to eradicate genetically or epigenetically defective male germ cells that are developmentally incompetent (64). Considering that the cellular functions of pRB are not limited to cell cycle regulation, it is likely that the reason for germline ablation in pRB-KO*^Blimp1^* mice is due to failure of pRB-deficient gonocytes to pass this developmental quality control check-point.

Perinatal testicular development in *pRB*-KO*^Ddx4^* mice (*pRB* deleted in ED15.5-17 gonocytes) is apparently normal although the number of spermatogonia in the pre/peripubertal testis is higher than in control mice (52). Despite a previously established role for pRB in control of gonocyte cell cycle exit before birth (55), this is not due to extended proliferation of gonocytes and *pRB*-KO*^Ddx4^* mice are born with a normal number of germ cells (52), suggesting that conditional deletion of *pRB* at the time of gonocyte quiescence, does not disrupt this state. Notably, and in contrast to pRB-KO*^Blimp1^* mice, the GSC pool is formed in pRB-KO*^Ddx4^* mice because GFRa1-positive (GDNF family receptor alpha 1) cells are observed at the basement membrane of the seminiferous epithelium (52). These GSCs, however, show very limited, if any, self-renewal capacity (52, 54). Although it has not been studied whether the GSC niche forms properly in pRB-KO*^Ddx4^* mice, it is conceivable that inability to self-renew is likely GSC-intrinsic rather than due to extrinsic factors, such as lack of GDNF (glial cell line-derived neurotrophic factor) (36, 65). Hu et al. (2013) also report that pRB-KO*^Ddx4^* GSC exit from self-renewal is followed by expansion of progenitor-A_undiff_ population (52). This is supported by increased density of spermatogonia in peripubertal mice but suffers some limitations because the authors use PLZF (promyelocytic leukemia zinc finger) as a marker of A_undiff_, while PLZF is also expressed in differentiating spermatogonia (66, 67). Nonetheless, these data support that pRB regulates the timely and stage-dependent cell cycle entry and exit in spermatogonia.

If pRB is ablated in progenitor-A_undiff_ or differentiating spermatogonia, as in *pRB*-KO*^Ngn3^* and *pRB*-KO*^Stra8^* mice, there are no effects on male fertility, but the testes of respective mice are often cystic and have areas that are devoid of spermatogenic cells, and sometimes, as Yang et al. (2013) report, house cells with neoplastic features (54). These results suggest that the consequences of pRB-deficiency in differentiation-committed and differentiating germ cells and all subsequent spermatogenic cell types are significant but possibly compensated by other RB family members, as also suggested by **Figure 2**, given the continuation of qualitatively normal spermatogenesis in adult *pRB*-KO*^Ngn3^* and *pRB*-KO*^Stra8^* mice.

To summarize, the spermatogenic phenotype that germ cell-specific *pRB* deletion inflicts highly depends on the developmental stage where the deletion has been induced. While *pRB*-KO*^Blimp1^* mice are sterile, pRB-KO*^Ddx4^* mice undergo 1-2 rounds of spermatogenesis, and both *pRB*-KO*^Ngn3^* and *pRB*-KO*^Stra8^* mice display qualitatively normal spermatogenesis and are fertile (52–54). These data demonstrate that pRB is a critical regulator of formation and maintenance of GSCs but dispensable for spermatogenic differentiation of their progeny.

### RB-E2F3 in Sertoli Cell Maturation

In addition to germ cells, the developmental and functional consequences of pRB ablation have also been studied in Sertoli cells (44, 45). Sertoli cells are the only somatic cell type inside the seminiferous tubules and they are paramount to testicular development, function and spermatogenesis (68). It is considered that the number of Sertoli cells ultimately defines sperm production capacity because each Sertoli cell is able to support a finite number of spermatogenic cells (69). Therefore, any factors that impinge on Sertoli cell proliferation or apoptosis are a potential threat to male fertility. Sertoli cells are specified in the mouse XY gonad following the expression *Sry* (sex-determining region Y) and its most significant downstream target gene *Sox9* (SRY-box 9) at ED10.5-11 (61). Sertoli cells then coordinate the differentiation of all other testicular cell types, including the germ cells. Sertoli cells undergo maturation in pubertal testis involving polarization, formation of the blood-testis barrier, a profound change in the transcriptome/proteome and exit from the cell cycle. Notably, Sertoli cells of an adult mouse do not proliferate but are in a seemingly terminally-differentiated state. However, under specific *in vitro* circumstances they are able to resume the cell cycle (70, 71). As discussed in detail below, similar to many other somatic cells, but not germ cells, Sertoli cells fail to enter a functionally mature mitotically-quiescent state if lacking pRB (44, 45, 52, 60).

The consequences of Sertoli cell-specific ablation of pRB have been explored in three studies, all of which relied on Cre expression under *Anti-Müllerian hormone* promoter (*pRB*-KO*^Amh^*) resulting in pRB loss of function from ED14.5 (44, 45, 47). The studies show congruent results: pRB-deficiency leads to rapid testicular atrophy and male infertility. However, while the gene expression is misregulated at least as early as in PND10 testis, testicular development and onset of spermatogenesis in juvenile mice appear unaffected in *pRB*-KO*^Amh^* mice, and until 6-weeks of age there are no obvious changes in testicular histology (44, 45). Subsequently, the changes in the phenotype are fast and the male mice become infertile by early adulthood. The testicular phenotype of *pRB*-KO*^Amh^* mice also includes Sertoli cell vacuolization, sloughing of Sertoli cells and immature germ cells from the seminiferous epithelium, increased germ cell apoptosis and Leydig cell hyperplasia (44, 45, 47). Interestingly, this might present an evolutionarily conserved function of RB proteins given a comparable phenotype in *Drosophila* following somatic deletion of *Rbf* (a pRB homolog) (72, 73).

There are a few remarkable changes in pRB-ablated Sertoli cell physiology that have a common nominator: inability to mature functionally. First of all, while WT Sertoli cells exit the cell cycle at around PND15, *pRB*-KO*^Amh^* Sertoli cells continue to proliferate at a constant rate at least until PND30 (45). This is followed by increased apoptosis probably due to activation of p53 pathway, thus precluding tumor formation (44). While the likely reason for spermatogenic failure in these mice is complex and multifactorial [loss of BTB (blood-testis barrier) integrity, apoptosis of pRB-deficient Sertoli cells to nurture and maintain spermatogenic cells etc.] it has not been carefully studied at which step spermatogenesis fails and whether functional GSCs were sustained in adult *pRB*-KO*^Amh^* mice. Considering the critical cell-autonomous role of pRB in GSCs, as described above, this is an interesting question and would deserve further investigation. Given the timeline of male infertility in these mice [2-3 months (44) and 3-6 months (45)] it is conceivable that GSCs are able to undergo at least some rounds of self-renewal in these conditions. This is also supported by qPCR data showing reduced but measurable levels of GSC-associated transcripts: *Gfra1* and *Lin28 (* 47). However, *Pou5f1* (*Oct4*) expression is lost in *pRB*-KO*^Amh^* testis suggesting a loss of progenitor-A_undiff_, where *Pou5f1* expression is enriched (36).

Interestingly, according to Rotgers et al. (2014) the BTB first organizes normally in 6-week old *pRB*-KO*^Amh^* mice but then disintegrates (45). However, the data from Nalam et al. (2009) indicate that the BTB is not functional even at this early time point and allows the leakage of a tracer from the interstitium to the adluminal compartment of seminiferous tubules, an immune-privileged site in normal testis (44). These findings suggest that pRB is not developmentally needed for formation of the BTB but is indispensable for its integrity and maintenance, which is another sign of Sertoli cell dedifferentiation and immaturity in *pRB*-KO*^Amh^* mice. Interestingly, Sertoli cells deficient for pRB display seminiferous epithelial cyclic activity, as judged by fluctuation of androgen receptor (AR) staining intensity between seminiferous tubule cross-sections (47). AR is one of the proteins that are known to be expressed in Sertoli cells in a seminiferous epithelial stage-dependent manner with the highest levels in early-to-mid stages and lowest levels in late stages (74, 75).

In WT testis pRB interacts with E2F3, the only activator E2F that is expressed in Sertoli cells (45). *E2f3* gene encodes two isoforms, *E2f3a* and *E2f3b*, both of which are expressed in the mouse testis. Interestingly, while Sertoli cells also express p130, Rotgers et al. (2014) do not confirm an interaction between E2F3 and p130 in WT testis. In *pRB*-KO*^Amh^* testis, however, E2F3 is found to form a complex with both p130 and p107, whose expression is induced in pRB-deficient Sertoli cells (45). This is in agreement with previous findings showing that when pRB is absent p107 and p130 also bind to activator E2Fs (E2F1-3), not just the repressors (E2F4-5) (76). While these findings imply that there might be a compensatory mechanism, the severity of the phenotype indicates that such a mechanism does not have any functional relevance. Already in their original paper Rotgers et al. (2014) provide data showing that impairment of spermatogenesis due to pRB-deficiency is attenuated by simultaneous silencing of *E2f3 in vivo (* 45). These results were corroborated by a follow-up study where *pRB-E2f3* compound-KO*^Amh^* mice were created (47). These mice display full spermatogenesis but interestingly the litter size is markedly reduced when compared to control. This might be due to disturbance of non-canonical, *i.e.* non-E2F-mediated, pRB functions in compound-KO*^Amh^* mice, including chromatin organization and nuclear architecture which are pronouncedly relevant in the spermatogenic context (77, 78). While spermatogenesis is qualitatively normal in *pRB*-KO*^Amh^*/*E2f3*-haploinsufficient mice, they display age-dependent testicular atrophy, and continuously cycling Sertoli cells, suggesting that *E2f3* gene dosage is critical for spermatogenesis. Notably, adult Sertoli cells of *pRB-E2f3* compound-KO*^Amh^* mice are non-proliferative and seem to be functionally comparable to their WT counterparts (47). In summary, these data show that spermatogenic failure, prolonged proliferation, increased apoptosis and dedifferentiation of Sertoli cells are mediated by adverse and non-restricted action of E2F3 in Sertoli cells of *pRB*-KO*^Amh^* mice, and highlights the role of pRB as a key regulator in life-long maintenance of the non-renewable Sertoli cell population.

**Figure 6:**
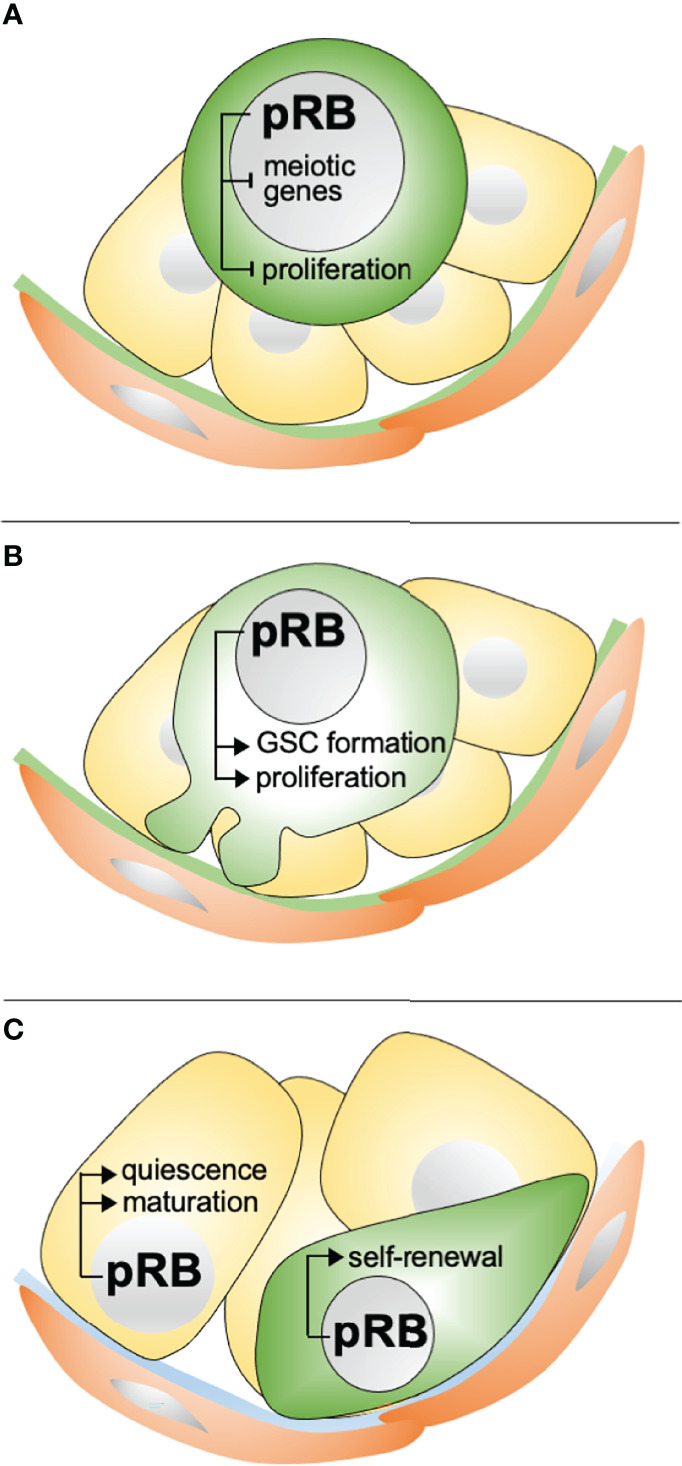
Known developmental roles of pRB in the testis. **(A)** In ED14.5-16.5 gonocytes, pRB is needed for cessation of proliferation and entry into a mitotically quiescent state. pRB likely also inhibits the expression of meiotic genes. **(B)** In the perinatal testis, pRB is needed for cell cycle re-entry and formation of the GSC pool. **(C)** In pubertal and adult testis, pRB is required for functional maturation of Sertoli cells, including their exit from the cell cycle, and self-renewal of GSCs.

Considering the spermatogenic rescue experiments by simultaneous deletion (47) or shRNA-mediated *in vivo* knock-down of *E2f3 (* 45) on a *pRB*-KO*^Amh^* background, it is surprising that *E2f3*-KO*^Amh^* mice, where *E2f3* has been deleted from fetal Sertoli cells, are fertile and show no signs of impaired spermatogenesis (47). The authors hypothesize that E2F4 might fully compensate for lack of E2F3 in these mice. While this might be possible there are, however, at least two obvious pitfalls in this interpretation. Firstly, E2F4 is considered a transcriptional repressor, whereas E2F3a isoform is classically considered a transcriptional activator. Secondly, E2F4 primarily associates with p107 and p130, not with pRB. Thus, further investigations are warranted to address this question. Results from these studies further support the concept that Sertoli cells are not terminally-differentiated but in a state of continuous cell cycle repression (79), and show that pRB is responsible for maintaining this non-proliferative state. These conclusions, however, suffer some limitations because pRB is ablated already in fetal Sertoli cells. An inducible *pRB*-deletion in adult Sertoli cells would be required to further elucidate this question. The developmental roles of pRB in the testis are summarized in **Figure 6**.

### pRB Is Not Involved in Testicular Tumorigenesis

To our knowledge, there are only very limited data available supporting a relationship between pRB-deficiency and increased tumorigenesis in the testis. Targeted deletion of *pRB* from prenatal germ cells results in germline ablation and infertility (44, 53, 54), and focal GCNIS-like (germ cell neoplasia in situ) like cells are only observed when *pRB* is deleted from progenitors and/or differentiating spermatogonia, arguably due to inability to commit to the spermatogenic lineage (54), an interpretation that might have some merit given that progenitor-A_undiff_ are enriched for pluripotency-associated transcripts (36). Ablation of pRB in Sertoli cells leads to continued engagement in the cell cycle but also increased apoptosis thus precluding tumor formation (44). Interestingly, Nalam et al. (2010) also studied the compound effects of *pRB* ablation on an inhibin-α-knockout (*Inha* KO) background (80). *Inha* KO mice are known to develop gonadotropin-dependent gonadal stromal tumors, which originate from Sertoli cells (81). Unexpectedly, the double-KO mice did not display exacerbation of the tumorigenic phenotype but Sertoli cell dysfunction took place earlier in these mice when compared to *pRB*-KO*^Amh^* mice (80). In summary, there are no strong indications that pRB would have tumor suppressor properties in the mouse testis.

### E2F1 Has a Multifaceted Role in the Testis

Similar to other tissues and cellular contexts, E2F1's role in the testis has deserved most attention within the E2F family. E2F1 transcription factor is a critical regulator of the cell cycle, and a direct target of pRB. However, like all activator E2Fs, E2F1 in particular displays intriguing functional dichotomy and is able to induce both proliferation and apoptosis (13). This is apparent also in the testis. E2F1 has been associated with progressive degeneration of the seminiferous epithelium (43, 82–84), GSC maintenance (43, 84), apoptosis of germ cells (43, 85) and Sertoli cells (44), germ cell neoplasia *in situ (* 86), testicular tumors (82), testicular descent in human (87, 88) and mouse (84), and human male infertility (87, 88).

Our understanding of E2F1's testicular function comes primarily from mouse work and use of *E2f1^tm1Meg^* mouse strain (89) (referred to *E2f1*-null hereafter). *E2f1-*null mice display characteristics of progressive testicular atrophy that is manifested in a background strain-dependent manner implying that genetic background of the mice has an impact on the time course and severity of the phenotype (43, 82–84, 89). Interestingly, on C57Bl/6 background the first signs of spermatogenic impairment can be observed as early as 20 days of age when the number of meiotic cells is reduced in *E2f1-*null mice when compared to control (43). E2F1 is an early regulator of the spermatogonial compartment and likely promotes spermatogonial apoptosis during the first wave of spermatogenesis (43). Somewhat counterintuitively, loss of E2F1 does not result in accumulation of spermatogonia during steady-state spermatogenesis but their gradual depletion and occurrence of tubules with Sertoli-cell-only phenotype (43, 82, 84). While the underlying mechanisms are not fully understood it is likely that GSCs in *E2f1-*null mice are lost *via* loss of self-renewal capacity and escape to differentiation. The phenotype thus shares remarkable but limited resemblance to pRB-KO*^Ddx4^* mice (52). While *E2f1*-null males remain fertile for at least 5-6 months (43, 82, 84), pRB-KO*^Ddx4^* mice become infertile by 2-3 months (52, 54). This is a significant difference because it shows that GSCs are able to self-renew in *E2f1*-null but not in pRB-KO*^Ddx4^* mice.

While the exact explanation for the discrepancy in time course is yet to be discovered, there are a couple of likely explanations: compensation by other E2F activators and non-E2F-mediated effects of pRB (discussed above). Notably, apart from *E2f1* no other *E2f*s were differentially expressed in *E2f1-*null testis casting doubts for compensation at the transcriptional level (43), yet leaving the door open for post-transcriptional mechanisms. Interestingly, when human E2F1 is overexpressed in the mouse testis, increased germ cell apoptosis is observed, accompanied with accumulation of undifferentiated spermatogonia (85), thus parallelling GDNF or NANOS2 overexpression phenotype (65, 90). Together these data demonstrate that E2F1 is a critical regulator of GSC maintenance and germ cell apoptosis. Although the definitive data are missing, as how E2F1 might be involved in GSC fate decisions, the literature provides some clues about the potential mechanisms. Transcriptomic changes in *E2f1*-null testis have been studied at 20 days of age by Rotgers et al. (2015) (43), and 3 and 7 months of age by Jorgez et al. (2021) (84). However, choice of the time point and method of study (microarray vs. qPCR) makes the results of these studies hard to compare. While the histological differences in a 20-day-old *E2f1*-null and WT mouse testis are relatively small making comparison between the genotypes feasible at this time point, the transcriptome at this age is different from adult testis with full spermatogenesis which complicates the comparison between the time points. Then again, the cellularity already in a 3-month-old and particularly in a 7-month-old *E2f1*-null testis is dramatically different from WT testis thereby complicating the analysis. For these reasons we will look at the findings separately.

Rotgers et al. (2015) identify a couple of E2F1 candidate target genes in a 20-day old mouse testis that, based on available studies, might be relevant for the development of the phenotype: *Cnot1 (CCR4-NOT Transcription Complex Subunit 1)* and *Chd1* (Chromodomain Helicase DNA Binding Protein 1) both of which are downregulated in *E2f1*-null testes (43). CNOT1 is particularly interesting because it directly interacts with NANOS2, a key intrinsic regulator of the male germline (90, 91). NANOS2 is involved in both degradation and sequestration of specific mRNA molecules in ribonucleoprotein complexes, and its loss results in GSC depletion and rapid germline ablation (59, 90–93). CNOT1 mediates NANOS2 interaction with CCR4-NOT, a major cytoplasmic deadenylase that primes mRNAs for degradation (91, 93). Importantly, CCR4-NOT–CNOT1–NANOS2 interaction is critical for NANOS2 function and maintenance of the male germline (91), a plausible mechanism also explaining GSC depletion in *E2f1*-null mice. Another interesting E2F1-candidate gene discovered by the microarray analysis is *Chd1*. While its role in spermatogenesis has not been studied, two of its family members CHD4 and CHD5 are critical for sperm production, albeit at the opposite ends of this complex process. CHD4 has been shown to be highly expressed in GSCs where its involved in their maintenance and self-renewal (94), whereas CDH5 is required for spermiogenesis and especially for chromatin condensation in elongating spermatids (95, 96).

Jorgez et al. (2021) studied the expression of select 66 mRNAs by qPCR and subsequent proteomic analyses (84). They found changes in the expression of cyclin genes and other E2F1 cell cycle targets. Interestingly, Wnt signaling pathway genes were differentially expressed between the genotypes, and all studied Wnt ligands were found generally upregulated, especially WNT4. Wnt signaling has been thought to prime GSCs for differentiation and promote exit from the self-renewing state (97–101). Boyer et al. (2012) reported that treatment of cultured GSCs with WNT4 reduces their stemness both *in vitro* and after transplantation (100). These data would support a model where GSCs in *E2f1*-null testis are subjected to differentiation-priming environment at the expense of self-renewal, thus resulting in their depletion over time. To explore this possibility Jorgez et al. (2021) generated a compound *E2f1*-null/*Wnt4*-null mouse line, where *Wnt4* was conditionally deleted from *Stra8*-expressing germ cells, that is progenitor-A_undiff_/differentiating spermatogonia. Remarkably, the spermatogenic capacity is qualitatively restored in these mice and they display nearly normal fertility parameters, demonstrating that many of the adverse effects of E2F1-deficiency can be overcome by simultaneous removal of WNT4. While the above findings provide a mechanism for GSC loss in *E2f1*-null mice, further studies are needed to address how the RB-E2F pathway is integrated in the control of GSC fate in WT mice, and how is it mechanistically linked with GDNF signaling (36, 65, 102) and mTOR (mammalian target of rapamycin) pathway (67, 103, 104) to balance self-renewal and differentiation of GSCs.

A typical feature of spermatogenic impairment when E2F1 is either deleted or over-expressed is spermatocyte apoptosis (43, 82, 85). While this leads to subfertility, *E2f1*-null male mice are able to sire viable offspring because some spermatocytes manage to avoid cell death. The reason for *E2f1*-null spermatocyte apoptosis is not known but there is data to support that DNA damage is not the underlying cause (43). Further studies are needed to unveil what induces spermatocyte apoptosis if E2F1 is either absent or overexpressed. Considering the expression pattern of E2F1 (highly expressed in premeiotic cells and early spermatocytes; **Figures 2**, **5**) and its role as a transcriptional activator, it is conceivable that the expression of E2F1-target genes needs to be delicately balanced for successful meiosis. It is likely that spermatocyte apoptosis is induced in a cell-intrinsic manner, also considering the fact that despite being expressed in peritubular cells and in the testicular interstitium, E2F1-deficiency was not found to affect testicular somatic cells or gonadotropin or androgen levels (43, 84). However, albeit not known to express E2F1, the Sertoli cells of *E2f1*-null mice display some transcriptomic changes when compared to controls. Surprisingly, while some of the changes may be explained by a change in cellularity in *E2f1*-null vs. control mice testis (43), misregulation of genes involved in tight and adherens junctions were shown to have functional consequences and the blood-testis barrier of 3-month-old *E2f1*-null mice was found leaky (84).

**Table 1** provides a summary of the spermatogenic phenotypes of different KO mouse strains where one or two genes encoding proteins of RB-E2F pathway has been deleted.

**Table 1:** Summary of spermatogenic phenotypes in different mouse strains with a deletion in gene(s) encoding RB-E2F pathway proteins.

Mouse strain	Time of deletion	Lineage where deleted	Normal establisment of the GSC pool	Qualitatively/Quantitatively normal 1^st^ round of spermatogenesis	Qualitatively/Quantitatively normal spermatogenesis in adulthood	Ref
*pRB*-KO*^Blimp1^*	ED6.5	germline	No	No/No	No/No	(53)
*pRB*-KO*^Ddx4^*	ED15.5-17	germline	n.a.	Yes/No	No/No	(52, 54)
*pRB*-KO*^Ngn3^*	postnatal	germline	Yes	Yes/n.a	Yes/No	(54)
*pRB*-KO*^Stra8^*	postnatal	germline	Yes	Yes/n.a	Yes/No	(54)
*pRB*-KO*^Amh^*	ED14.5	Sertoli	Yes	Yes/Yes	No/No	(44, 45)
*E2f3*-KO*^Amh^*	ED14.5	Sertoli	Yes	Yes/Yes	Yes/Yes	(47)
*pRB*-KO*^Amh/^E2f3*-KO*^Amh^*	ED14.5	Sertoli	Yes	Yes/n.a	Yes/No	(47)
*pRB*-KO*^Amh/^E2f3^+/-^*	ED14.5	Sertoli	Yes	Yes/n.a	Yes/No	(47)
*E2f1^tm1Meg^*	zygotic	universal	Yes	Yes/No	No/No	(43, 84)
*E2f1^tm1Meg^/Wnt4*-KO*^Stra8^*	zygotic/ postnatal	universal/ germline	Yes	Yes/n.a	Yes/No	(84)

n.a, not analyzed.

### Repressor E2Fs E2F4 and E2F5 Are Needed for the Development of Male Reproductive Tract

Compared to activator E2Fs, the functions of repressor E2Fs have been less investigated. While both *E2f4* and *E2f5*-deficient mice are viable, they have a shortened lifespan (105–107). *E2f4-*mutants display developmental defects in multiple tissues and have a high early postnatal lethality due to susceptibility to infections (106, 107). *E2f4*-deficient male mice were found subfertile/infertile although the histology of male reproductive organs was reported normal (106, 107), and the underlying basis for this observation therefore remains elusive. There is a lack of knowledge concerning fertility of *E2f5*-mutant mice. However, *e2f5* mutation in the zebrafish results in male infertility due to a spermatogenic arrest in prophase I, and subsequent apoptosis of spermatocytes (108). Rotgers et al. (2014, 2019) report a clear decrease in *E2f4* and *E2f5* expression in *pRB*-KO*^Amh^* mouse testis which is, however, likely due to lack of meiotic and postmeiotic germ cells (cf. **Figure 2**). Interestingly, E2F4 and E2F5 have been shown to display redundant roles in controlling the development of the male reproductive tract. *E2f4*-deficiency within the efferent ducts on a *E2f5* heterozygous background leads to a loss of multiciliated cells from the efferent ducts, dilation of the seminiferous tubules and the rete testis, and infertility.

The other repressor E2Fs have been even less studied in the testis. *E2f6*-deficient mice are born at an expected Mendelian frequency, are viable and fertile, and grow and develop normally (109). Pohlers et al. (2005) have reported that E2F6 is needed to suppress the expression of germline genes in somatic tissues (109). However, the significance or functions of E2F6 in the germline cells are not known but mRNA level analysis (**Figure 2**) suggests they might be subtle. No fertility problems have been reported in mice deficient for *E2f7* or *E2f8*, whereas the double-null mice are early embryonically lethal precluding any analysis on germline effects (110).

## Concluding Remarks

Considering its role as the gatekeeper of cell cycle, it is no wonder that the RB-E2F pathway is critically important for development and function of the testis. Both the germline and the somatic Sertoli cells depend on proper regulation of its activity during formation of the testis and under steady-state spermatogenesis. Continuous production of sperm relies on lifelong maintenance of GSCs and functionally mature Sertoli cells, two fundamental outcomes of normal function of RB-E2F pathway, highlighting its importance for male fertility. Despite the fact that the functional consequences of pRB, E2F1 and E2F3-deficiency in the testis have been relatively well characterized, further studies are warranted to elucidate how RB-E2F is integrated into the regulation of the germline at the mechanistic level plus shed light on the diversity of RB-E2F signaling beyond pRB and E2F1 and E2F3. Notably, despite relatively high level of mRNA expression in germ cells, the spermatogenic functions of p107, p130, E2F4 and E2F5 are virtually undefined. Examining their roles would probably require creation of compound inducible KO mouse models to circumvent functional redundancy within both the RB and E2F families, and for targeted analyses of spermatogenic functions. Their temporally restricted patterns of expression along the spermatogenic trajectory are intriguing and worthy of deeper investigation.

## Author Contributions

J-AM and JT: Conception of the article, writing and editing. All authors contributed to the article and approved the submitted version.

## Conflict of Interest

The authors declare that the research was conducted in the absence of any commercial or financial relationships that could be construed as a potential conflict of interest.

## Publisher’s Note

All claims expressed in this article are solely those of the authors and do not necessarily represent those of their affiliated organizations, or those of the publisher, the editors and the reviewers. Any product that may be evaluated in this article, or claim that may be made by its manufacturer, is not guaranteed or endorsed by the publisher.
